# A Prospective Repurposing of Dantrolene as a Multitarget Agent for Alzheimer’s Disease

**DOI:** 10.3390/molecules24234298

**Published:** 2019-11-25

**Authors:** Isabella Bolognino, Nicola Giangregorio, Leonardo Pisani, Modesto de Candia, Rosa Purgatorio, Annamaria Tonazzi, Cosimo Damiano Altomare, Saverio Cellamare, Marco Catto

**Affiliations:** 1 Department of Pharmacy-Drug Sciences, University of Bari Aldo Moro, Via E. Orabona 4, 70125 Bari, Italy; isabella.bolognino@uniba.it (I.B.); leonardo.pisani@uniba.it (L.P.); modesto.decandia@uniba.it (M.d.C.); rosa.purgatorio@uniba.it (R.P.); cosimodamiano.altomare@uniba.it (C.D.A.); saverio.cellamare@uniba.it (S.C.); 2 Institute of Biomembranes, Bioenergetics and Molecular Biotechnologies (IBIOM), National Research Council (CNR), via Amendola 122/O, 70126 Bari, Italy; n.giangregorio@ibiom.cnr.it (N.G.); a.tonazzi@ibiom.cnr.it (A.T.)

**Keywords:** dantrolene, drug repurposing, multitarget activity, neuroprotection, carnitine/acylcarnitine carrier, Alzheimer’s disease

## Abstract

The orphan drug dantrolene (DAN) is the only therapeutic treatment for malignant hyperthermia (MH), a pharmacogenetic pathology affecting 0.2 over 10,000 people in the EU. It acts by inhibiting ryanodine receptors, which are responsible for calcium recruitment in striatal muscles and brain. Because of its involvement in calcium homeostasis, DAN has been successfully investigated for its potential as neuroprotecting small molecule in several animal models of Alzheimer’s disease (AD). Nevertheless, its effects at a molecular level, namely on putative targets involved in neurodegeneration, are still scarcely known. Herein, we present a prospective study on repurposing of DAN involving, besides the well-known calcium antagonism, inhibition of monoamine oxidase B and acetylcholinesterase, cytoprotection from oxidative insult, and activation of carnitine/acylcarnitine carrier, as concurring biological activities responsible for neuroprotection.

## 1. Introduction

**Figure 1:**
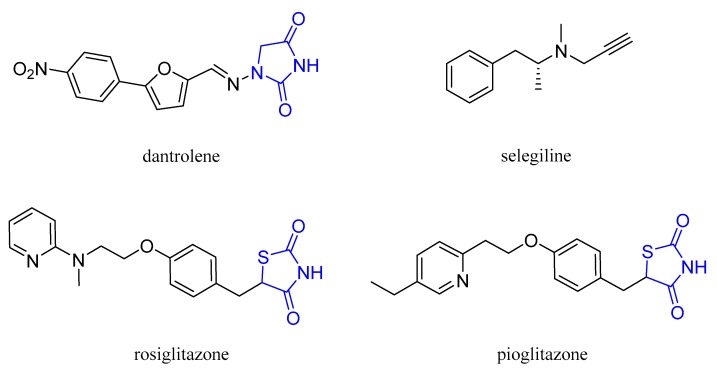
Structures of dantrolene and monoamine oxidase (MAO) inhibitors.

Dantrolene (DAN), see Figure 1, a lipophilic hydantoin derivative, was synthesized for the first time in 1967 by Snyder and coworkers who soon discovered its muscle-relaxing properties [1]. DAN is the only drug approved for treating malignant hyperthermia (MH), that is, a pharmacogenetic disorder triggered by volatile anesthetics and characterized by muscle rigidity and fatal rise in body temperature [2]. In a MH episode, an excessive leak of Ca^2+^ ions from sarcoplasmic reticulum (SR) results in an uncontrolled hypermetabolism that leads to respiratory and metabolic acidosis due to rapid consumption of energy stores and ATP [3].

DAN depresses the intrinsic mechanism of excitation–contraction coupling by acting on the ryanodine subtype 1 receptor (RyR1) within the SR of muscle cells [4]. In addition to RyR1, other two forms of these receptors are known, i.e., RyR2, mainly expressed in cardiac muscle, and RyR3, which is particularly abundant in the brain [5]. DAN also reduces neuronal cell death induced by both transient ischemia and hypoxia and glucose deprivation in rats [6,7]. Nowadays, intravenous DAN is successfully used to treat 3,4-methylenedioxymethamphetamine (MDMA, ecstasy) intoxication [8]. In this respect, some authors showed that a neurotoxic dose of MDMA in rats triggers a mitochondrial oxidative damage in brain regions, increases the lipid and protein peroxidation, and induces mitochondrial DNA deletion [9]. Furthermore, the activity of monoamine oxidases A and B (MAO A and B) and the resulting production of hydrogen peroxide as byproduct of MAO catabolic activity are increased by MDMA-induced release of neurotransmitters. A proof of this is the capability of selegiline, as seen in Figure 1, a selective MAO B inhibitor, in preventing most of oxidative stress damages induced by hydrogen peroxide and related reactive oxygen species (ROS) in MDMA intoxication [10].

The pivotal role of MAOs as metabolizing enzymes in modulating the concentration of neurotransmitters has attracted significant attention as favored target in some severe and chronic neurodegenerative diseases [11,12]. This well-established reputation is closely related to the substrate and tissue specificity of both isoforms: MAO A selective inhibitors are clinically administered as antidepressants [13], while MAO B selective inhibition is typically used for the treatment of the early symptoms of Parkinson’s disease (PD) [14]. Recent studies demonstrated the involvement of both isoforms into the pathogenesis and progression of heart failure [15]. The neuroprotective effects of MAO B inhibitors also provide the rationale for their potential in Alzheimer’s disease (AD), particularly for the efficacy of MAO B inhibition in reducing ROS toxicity and oxidative stress (OS) [16].

The multifactorial nature of AD and the absence of an effective and long-lasting therapy so far, have stimulated medicinal chemists to follow multitarget drug design strategies based even on repositioning of approved drugs [17]. A recent example is represented by the antidiabetic drugs rosiglitazone and pioglitazone, as seen in Figure 1, isosteric analogues of hydantoins [18] that competitively inhibit MAO B with a low- and submicromolar K_i_ value, respectively, for the human enzyme. The high specificity shown by pioglitazone, and the crystallographic data in support of it [19], justify the possible repositioning of these molecules among promising neuroprotective agents.

In addition to MAO inhibition, drugs able to restore appropriate neurotransmitter (namely acetylcholine, ACh) levels found their rationale against AD in the so-called cholinergic theory addressing the inhibition of acetylcholinesterase (AChE) [20]. Albeit most clinically administered AChE inhibitors (AChEIs) show limited or palliative care, research is fervent and interesting perspectives are emerging [21]. In this context, dual-targeting compounds inhibiting AChE and MAOs may significantly slow down the progression of AD, in addition to mitigating its symptoms [22,23].

The main biochemical property of DAN in inhibiting the calcium release by RyRs suggests possible interactions with other transport systems present at a cellular and subcellular level. RyR1 has been described as a redox-sensitive system, for which reversible redox modifications of specific reactive cysteine residues (*S*-glutathionylation, *S*-nitrosylation) or their oxidation to form disulfide bridges, give rise to the active conformation responsible for calcium influx [24]. RyR1 shares this redox sensitization with many membrane transporters. We pointed at the study of DAN on carnitine/acylcarnitine carrier (CAC), an antiport shuttle localized at the inner mitochondrial membrane, involved in the trafficking of acylcarnitines into the mitochondrion for the synthesis of acetyl-CoA by β-oxidation pathway and ATP production [25,26]. CAC is also known to play a key role in the transport of acetyl-l-carnitine (ALC), a well-recognized nutraceutical [27].

On this basis, we decided to investigate these off-target activities of DAN, not only in inhibiting MAO and AChE, but also in modulating CAC activity. The neighboring proximity of MAO and CAC within the mitochondrial membranes suggests that inhibition of the former, hampering the production of ROS, may indirectly prevent the inactivation of the latter. Besides, a possible role of endogenous ALC exported by CAC in OS conditions has been discussed. Although DAN does not appear at a glance as a “classical” reducing agent, its antioxidant properties have been described [28]. Thus, in vitro antioxidant potency was tested with DPPH and ABTS radical scavenging assays. To deepen this feature, we also tested DAN in a cell-based method using immortalized SH-SY5Y neuroblastoma cells in OS conditions induced by dopamine.

## 2. Results and Discussion

### 2.1. Stability of Dantrolene in Buffered Solutions

In order to check the hydrolytic stability of DAN in buffered media, we used a HPLC method to check the stability of 20 μM DAN in phosphate buffered saline (PBS) at physiological pH of 7.4, at 37 °C. DAN was fully stable, even in prolonged incubations (92% of recovery after 60 h). The same result was obtained for shorter (2 h) incubation times in phosphate pH 7.4 and PIPES pH 7.0 buffers.

### 2.2. Inhibition of MAOs and ChEs

**Figure 2:**
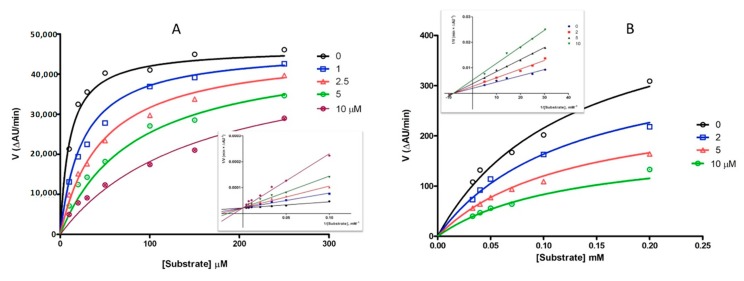
Michaelis–Menten curves of inhibition kinetics of dantrolene: (**A**) hMAO B (substrate: kynuramine); (**B**) hAChE (substrate: acetylthiocoline). Insets: Lineweaver–Burk plots. Figures represent a single experiment.

Routine kynuramine fluorescence [29] and Ellman’s spectrophotometric [30,31] assays were used for in vitro inhibition of human MAO B (hMAO B) and acetylcholinesterase (hAChE), respectively. Selectivity over the corresponding isoforms hMAO A and butyrylcholinesterase (hBChE) was also verified. Data in Table 1 show good inhibition values of hMAO B and hAChE in the low micromolar range, with fair (MAO B vs. A) or high (AChE vs. BChE) selectivity. The investigation of inhibition kinetics of DAN resulted in Michaelis–Menten curves fitting for competitive (hMAO B) and noncompetitive (hAChE) inhibition, as confirmed by Lineweaver–Burk double-reciprocal plots, as seen in Figure 2. This result provided the proof of a noncovalent, reversible occupancy of DAN within the catalytic cavity of MAO B, as already described for glitazone derivatives [19]. In turn, the potency of AChE inhibition was seven-fold lower than reference compound galantamine; the noncompetitive inhibitory mechanism on hAChE may lie in an interaction limited to the peripheral binding site of the enzyme, without interference with the catalytic site.

### 2.3. Inhibition of Self-Aggregation of Amyloid Peptides

**Figure 3:**
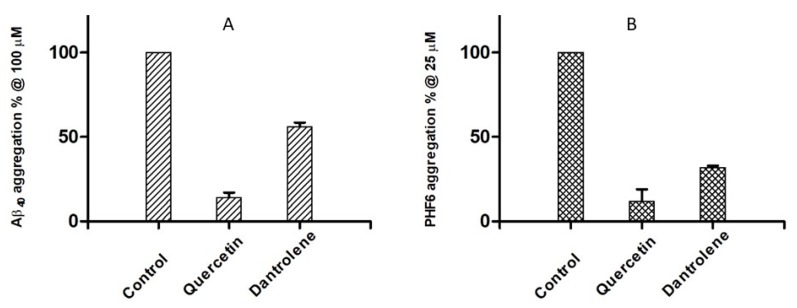
Inhibition of self-aggregation of Aβ_40_ 30 μM (**A**) and PHF6 50 μM (**B**) by dantrolene and the reference inhibitor, quercetin.

As an additional proof of the potential of DAN in interfering with AD-related processes, we investigated the effects on the in vitro self-aggregation of beta amyloid peptide 1–40 (Aβ_40_) and Tau hexapeptide (306)VQIVYK(311) (PHF6). Aβ_40_ is used as an in vitro probe of the fibrillization process leading to amyloid fibrils and ultimately to senile plaques in AD [32], while PHF6 represents the highly proaggregating sequence of the repeated domain R3 of Tau protein, responsible of the formation of neurofibrillary tangles and other tauopathies in NDs [33]. With this aim, we developed a fast screening method of Aβ_40_ aggregation using thioflavin T (ThT) as fluorescent probe and 2% 1,1,1,3,3,3-hexafluoro-2-propanol (HFIP) as aggregation enhancer [34]. Regarding PHF6, we started from an already published methodology [35] for the setup of an in-house method, with 2,2,2-trifluoroethanol (TFE) as the cosolvent. Results in Figure 3 highlight a good inhibitory potency of DAN of 56% and 32% of aggregation of Aβ_40_ and PHF6, respectively. Quercetin was used as a standard reference for the inhibition of amyloid aggregation [36]. The concentrations of inhibitors were 100 μM and 25 μM in Aβ_40_ and in PHF6 assay, respectively. The high planarity and the extended conjugation of aromatic rings of DAN may represent a key feature for β-sheet intercalation and disruption, while the heteroatom-rich hydantoin core may behave as hydrogen bond (HB) donor/acceptor for strong HB and dipolar interactions with the peptide backbone [37].

### 2.4. Radical Scavenging Activity

**Figure 4:**
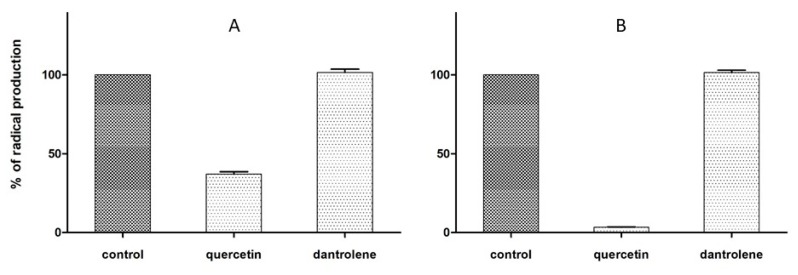
Antioxidant effects of dantrolene and the reference, quercetin, in scavenging of 100 μM DPPH (**A**) and ABTS (**B**) radical species. Values are mean ± SD for compounds at a concentration of 50 μM (DPPH assay) or 0.2·[ABTS^+^].

Previous studies [28] assessed a high antioxidant potency of DAN in inhibiting the Fe^2+^/thiocyanate peroxidation of linoleic acid and in Fe^3+^/Fe^2+^ reduction. Starting from these evidences, we tested DAN in two in vitro models of radical scavenging activity, i.e., the 1,1-diphenyl-2-picryl-hydrazyl (DPPH) and 2,2′-azinobis-(3-ethylbenzothiazoline-6-sulfonic acid) (ABTS) free radical production [38]. Both generate stable nitrogen radicals showing high absorption in visible spectrum, thus allowing easy and straightforward spectrophotometric measures. Results showed no activity of DAN in both assays, in contrast with quercetin, which behaved as a strong radical scavenger, as is typical for polyphenols, as seen in Figure 4. It can be assumed that the antioxidant activity of DAN is independent from its radical scavenging properties.

### 2.5. Cell-Based Assay of Neuroprotection

**Figure 5:**
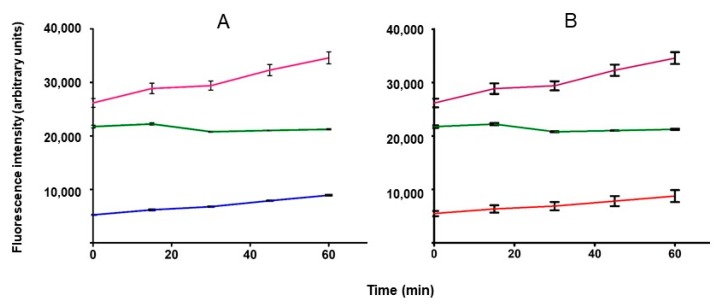
Dopamine-induced oxidative insult in SH-SY5Y cells; DCF-DA assay. (**--**), Control cells; (**--**), dopamine 10 nM; (**A**) (**--**), dopamine 10 nM + dantrolene 100 nM; (**B**) (**--**) dopamine 10 nM + phenelzine 100 nM.

In order to get further information about the potential neuroprotective effects exerted by DAN, we tested it in a 2′,7′-dichlorofluorescin diacetate (DCF-DA) fluorescence-based assay, measuring the production of ROS induced by dopamine in cultured SH-SY5Y cells. Immortalized neuroblastoma cells are a widely used model for neuroprotection assays, while dopamine at a concentration of 10 nM acts as an inducer of oxidative stress [39], being metabolized by MAOs to form hydrogen peroxide [40]. Once hydrolyzed and oxidized, DCF acts as the fluorescing probe of the OS state of cells. In the presence of an antioxidant, or even of a MAO inhibitor, ROS burden is lowered and DCF fluorescence decreased. Figure 5A shows that DAN was very effective in reducing ROS oxidation of DCF. Its activity is completely superimposable to that of phenelzine, a nonselective MAO inhibitor used as a positive control in Figure 5B [41]. These results gave further proof that protection from ROS is exerted by DAN through inhibition of MAO (specifically MAO B), rather than its intrinsic antioxidant properties.

### 2.6. Activation of CAC Transport

CAC contains six cysteine residues whose structure–function relationships have been well studied by site-directed mutagenesis, chemical modification, and homology modelling. In particular, two of these residues, C136 and C155, play a regulatory functional role depending on the redox state of the protein. These amino acids represent the specific target for various physiological compounds involved in the cell redox homeostasis, such as GSH/GSSG, nitric oxide, and hydrogen sulphide, inhibiting or activating the transport activity of the carrier [42,43,44]. The formation of a disulfide bridge by oxygen exposure or in conditions of OS inhibits the transporter [45], indicating that the protein is active only when the cysteines are in the reduced form, and suggesting a role of the couple C136-C155 as redox sensor. Thus, it was tested whether the reducing action of DAN was effective in activating CAC, since this feature would represent an interesting intervention for restoring abnormal depletion of ATP and increasing cellular levels of ALC.

**Figure 6:**
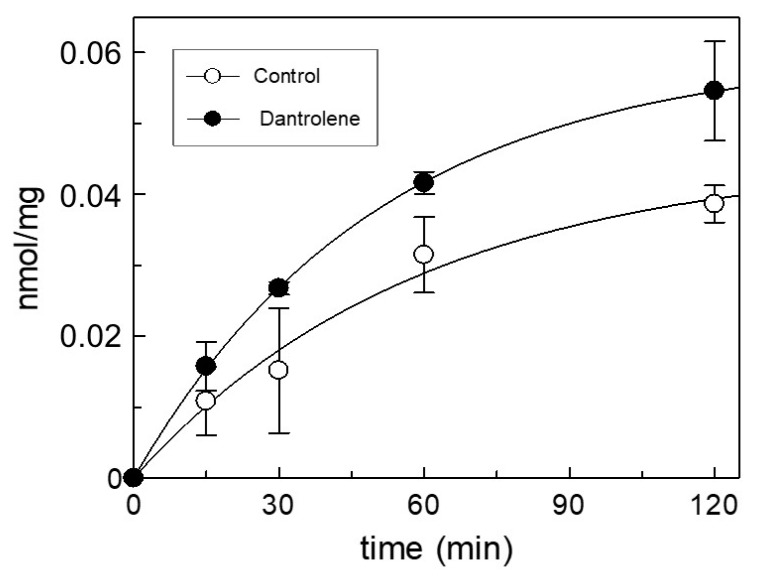
Time course of [^3^H]-carnitine uptake within intact mitochondria. The plot displays the curves of control and treated mitochondria by 50 μM dantrolene sodium. The data represent means ± SD of at least three independent experiments.

First, it was verified whether DAN entered intact mitochondria and had an effect on the CAC function. The purification of crude rat mitochondria and the measurement of CAC transport activity are described in Materials and Methods. The uptake of carnitine was started by adding 0.05 mM [^3^H]-carnitine in the presence or absence of 50 μM DAN (dissolved in 1:1 DMSO/H_2_O). The transport activity was inhibited at various times by adding cold 1 mM *N*-ethylmaleimide (NEM) in a buffer containing 150 mM sucrose, 50 mM TRIS-HCl, 50 mM KCl. Figure 6 shows the time-dependent antiport activity in mitochondria incubated with DAN or buffer alone. Addition of DAN to intact mitochondria induced an increase of transport function up to 41% after 120 min.

**Figure 7:**
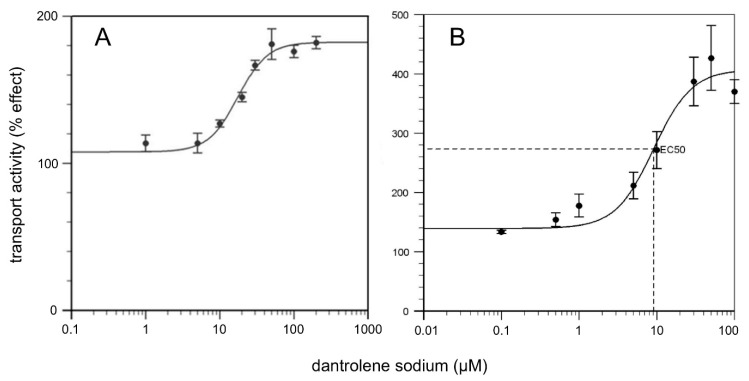
Dose–response curves of activation of CAC extracted from mitochondria (**A**) and purified recombinant WT protein (**B**). The antiport rate was measured by adding 0.1 mM [^3^H]-carnitine to proteoliposomes containing 15 mM internal carnitine and stopped after 30 min. Dantrolene sodium (DAN), at the indicated concentrations, was added 2 min before the transport assay. Percentage of effect on the transport activities are reported. The values are means ± SD from three independent experiments.

To detect the effect of DAN on native CAC, mitochondrial extract was reconstituted into liposomes and transport activity was measured adding various concentrations of compound. In Figure 7A, the dose–response curve is shown. To calculate the EC_50_ value, that is, the concentration which increases the transport activity of the carrier by 50% compared to the control, a wide concentration range (1–200 μM) was studied. The EC_50_ measured after 30 min of incubation was 18 ± 5 μM, a concentration close to that found in the cytosolic compartment in vivo (10 μM) during the therapeutic treatment [46,47]. Thus, the effect of different concentrations of DAN was tested also on the purified recombinant wild-type (WT) CAC carrier reconstituted into liposomes, measuring the antiport rate of carnitine. The EC_50_ derived from the dose–response curve after 30 min of incubation was 9.3 ± 3.1 μM, whereas the whole activation of the WT protein was observed at concentrations above 50 μM, as seen in Figure 7B. These data confirm that CAC is a specific target of DAN.

**Figure 8:**
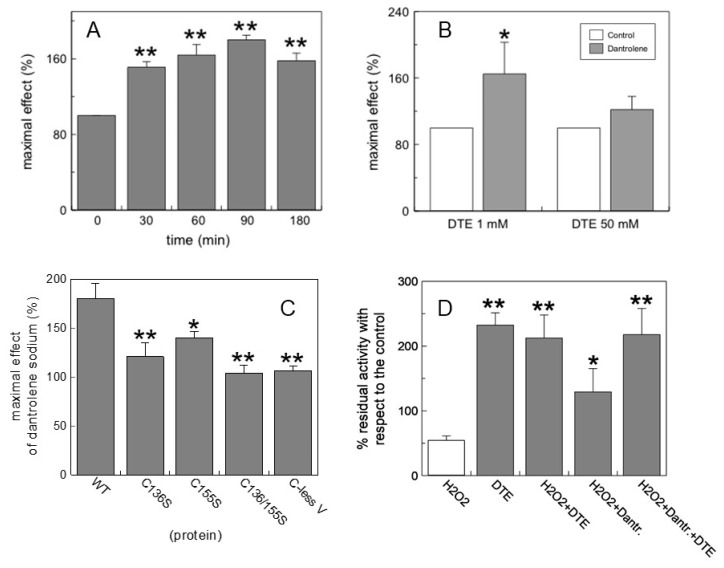
Effects of dantrolene on CAC carrier. (**A**) Time-dependent activation of CAC by DAN. (**B**) activation of CAC by DAN in different reducing conditions. (**C**) Comparison of activation by DAN of WT CAC and some critical cysteine-modified mutants. (**D**) Recovery of transport activity of CAC in oxidative conditions (hydrogen peroxide 1 mM). Concentrations of DAN: A–C, 10 μM; D, 20 μM. The antiport rate was measured adding 0.1 mM [^3^H]-carnitine to proteoliposomes containing 15 mM internal carnitine and stopped after 30 min. The values are means ± SD from three independent experiments. Significant difference from the controls, as calculated from Student′s *t*-test analysis, is marked by * *p* < 0.05 and ** *p* < 0.01.

To test the stability of DAN during its incubation with CAC and then calculate the maximum efficacy over time on the protein, transport activity of WT was measured at time intervals with 10 μM DAN, as seen in Figure 8A. The graph displays how the action of DAN remains almost constant over time with average values of around 160% compared to the control. These data may indicate that the stability of DAN may allow the molecule to cross mitochondrial membranes and reach the carnitine/acylcarnitine carrier.

The reducing properties of DAN were studied on the recombinant WT CAC with the protein at different states of oxidation. Figure 8B highlights that when the carrier was incubated during the transport activity with 1 mM dithioerythritol (DTE), 10 µM DAN was able to reduce a major aliquot of oxidized protein (70% recovery of activity compared to the control). When the carrier was incubated with 50 mM DTE, recovery was 25%, demonstrating that the action of DAN is exerted on thiol groups. To identify the cysteines responsible for the activation of the CAC carrier, the proteoliposomes of the WT protein and the mutants C136S, C155S, C136/155S, and C-lessV (C23V/C58V/C89S/C136V/C155V/C283S) were incubated with 10 μM DAN. The increase in transport activity observed on WT (180%) was significantly lost when cysteines C136 and C155 were alternately or together mutated (C136S, C155S, and C136/155S) or all cysteine residues were replaced (C-less V), as seen in Figure 8C, indicating that these amino acids are the specific redox targets for DAN. Further evidence of the reducing action of DAN on CAC was obtained by oxidation of the reconstituted WT protein under controlled conditions, i.e., in the presence or absence of hydrogen peroxide and its subsequent degradation by the enzyme catalase [45]. Figure 8D shows that the transport activity decreased to about 50% of the untreated control by adding 1 mM H_2_O_2_ to proteoliposomes for 10 min at 37 °C. The addition of 20 µM DAN led to a significant recovery (about 80%) of the transport function, after treatment of the carrier with H_2_O_2_. These data suggest that DAN could reverse the oxidizing action of hydrogen peroxide on the CAC, re-establishing the entry of fatty acids, the activation of the β-oxidation, and therefore the production of ATP.

Although ALC is able to cross the blood–brain barrier [48] and provide neuroprotection in the therapy of neurodegenerative diseases [49], even in various conditions of metabolic stress [50], the bioavailability of ALC in brain after oral administration is very low. The acetyl group of ALC is subjected to fast renal clearance, while in the gut and liver, it is converted in acetyl-CoA to allow acetylation reactions [51]. Since treatment with DAN may reactivate the function of CAC under OS conditions, the enhanced export of endogenous ALC produced during β-oxidation [27] could represent an important source of acetyl moieties, conferring effective cell neuroprotection.

## 3. Materials and Methods

All the chemicals, enzymes, solvents, and reagents were purchased from Sigma-Aldrich Europe (Milan, Italy) unless specified, and used without further purification.

### 3.1. Buffer Stability Assay

The analytical HPLC measurements were performed on a Waters 1525 HPLC System (Waters, Milan, Italy) equipped with a Waters 2587 variable-wavelength UV-Vis detector and a Waters 717 plus autosampler. The chromatographic data were acquired using the Waters Breeze software (version 3.20). Analyses were performed on a Phenomenex C18 column (150 × 4.6 mm i.d., 3 µm particle size; Phenomenex srl, Castel Maggiore, Italy) using a mobile phase of methanol/water (75:25 *v/v*), with aqueous formic acid 0.1%. The used flow rate was 0.5 mL/min and the injection volume was 20 µL. Wavelength UV-Vis detector was set at 380 nm.

The chemical stability of dantrolene sodium was evaluated in a phosphate buffered saline solution pH 7.4 (10 mM HPO_4_^2−^/H_2_PO_4_^−^, 100 mM NaCl) at 37 °C. Samples were tested in triplicate starting from three different stocks solution (1 mM), which were prepared separately.

### 3.2. Monoamine Oxidase Inhibition Assay

An already published protocol using human recombinant monoamine oxidases A (250 U/mg) and B (59 U/mg; microsomes from baculovirus infected insect cells) [29] was used by applying a 96-well plate technique (instrument Infinite M1000 Pro, Tecan, Cernusco s.N., Italy). Briefly, compounds at a concentration ranging from 30 to 0.01 μM were preincubated 20 min at 37 °C with substrate kynuramine, in phosphate buffer 8.0 made 0.39 osmolar with KCl. After addition of enzyme and further 30 min of incubation, NaOH was added and the fluorescence read at 310/400 excitation/emission wavelength. Experiments were performed in duplicates in black, flat-bottomed polystyrene 96-well microtiter plates (Greiner Bio-One GmbH, Frickenhausen, Germany). For inhibition kinetics, five concentrations of DAN (ranging from 0 to 10 μM), and seven concentrations of kynuramine (from 10 to 250 μM) were used. Inhibition values and kinetics were calculated by means of Prism (GraphPad Prism version 5.00 for Windows, GraphPad Software, San Diego, CA, USA).

### 3.3. Cholinesterase Inhibition Assays

An already published method was used [31] to apply the Ellman’s spectrophotometric assay [30] to the 96-well plate technique. Briefly, human recombinant AChE (2770 U/mg) or BChE from human serum (50 U/mg) were incubated in phosphate buffer pH 8.0 with test compounds (concentrations ranging from 30 to 0.01 μM) and 5,5′-dithiobis-(2-nitrobenzoic acid) (DTNB) as the chromophoric reagent. After 20 min of incubation at room temperature, substrate acetyl- or butyrylthiocoline was added and the increase of absorbance read at 412 nm within 5 min. Experiments were performed in duplicate in transparent, flat-bottomed polystyrene 96-well microtiter plates, and readings made with Tecan Infinite M1000 Pro instrument. For inhibition kinetics, four concentrations of DAN (ranging from 0 to 10 μM), and six concentrations of acetylthiocoline (from 33 to 200 μM) were used. Inhibition values and kinetics were calculated by means of Prism.

### 3.4. Inhibition Assay of β-Amyloid Aggregation

An already established assay [52] was used in a 96-well plate procedure, using HFIP as aggregation enhancer [34]. Briefly, test compounds (100 μM) were incubated with Aβ_40_ (30 μM) and 2% HFIP in PBS pH 7.4 for 2 h at room temperature. After addition of ThT, the fluorescence was read at 440/485 excitation/emission wavelength. Experiments were performed in triplicates in black low-binding, flat-bottomed polystyrene 96-well microtiter plates. Inhibition values were calculated by means of Prism.

### 3.5. Inhibition Assay of PHF6 Aggregation

Highly aggregating hexapeptide Ac-VQIVYK-NH_2_ (trifluoroacetate salt; JPT Peptide Technologies GmbH, Berlin, Germany) was used as mimic of tau fibrillization in vitro. We applied an already published screening method [35], using ThT as fluorescing probe of aggregation, and modified as follows. PHF6 was pretreated by dissolution to 1.5 mM concentration in TFE, standing overnight at room temperature. This solution was diluted three-fold with PBS before incubation assays. Incubation samples were prepared in black low-binding, flat-bottomed polystyrene 96-well microtiter plates, containing PHF6 (final conc. 50 μM), inhibitor (25 μM) and ThT (10 μM) in a final volume of 180 μL, and immediately read with Tecan Infinite M1000 Pro instrument. Samples were set in triplicate and their fluorescence values subtracted of the blank. Control samples (i.e., free aggregation of peptide) were prepared as the reference. Fluorescence was measured at 440/485 excitation/emission wavelength within 2 h at 25 °C. Endpoint fluorescence values (arbitrary fluorescence units, AFU) were used for the calculation of residual aggregation as in Equation (1):

% residual aggregation = 100 × AFU_sample_/AFU_control_ (1)

### 3.6. In Vitro Radical Scavenging Assays

Already published protocols for DPPH and ORAC radical scavenging were used [38,53]. The scavenging of the radical DPPH^·^, or the monocation radical ABTS^·+^, were determined by measuring the loss of absorbance at 515 or 755 nm, respectively. Samples were prepared in clear polystyrene flat-bottomed 96-well microplates. Briefly, for the DPPH assay, to 35 μL of test sample solutions (or vehicle for controls: ethanol containing 5% DMSO), 215 μL of ethanol solution of DPPH were added. The final concentrations of DPPH^·^ and inhibitors were 100 μM and 50 μM, respectively. To form ABTS^·+^, a 10 μM ABTS water solution was added to 8.2 mM potassium persulfate in water (ratio 7/3); this solution was stored to darkness for at least 16 h, then diluted 60-fold with ethanol immediately before use. A preliminary absorbance reading at 755 nm allowed to determine the actual ABTS^·+^ concentration (normally 60–70 μM; extinction coefficient ε = 12,000) and set the final concentration of inhibitors to 0.2[ABTS^·+^]. 215 μL of ABTS^·+^ solution were mixed with 35 μL of stock solution of inhibitors and the decrease in absorbance was monitored. Antioxidant activity was determined from Equation (2) as the percentage of radical production of inhibitor containing samples, compared with control:

% radical production = 100 × ABS_sample_/ABS_control_ (2)

### 3.7. Cell Culture and DCF-DA Assay

The cell line SH-SY5Y (obtained from the National Cancer Institute, Biological Testing Branch; Frederick, MD, USA) was maintained in the logarithmic phase at 37 °C in a 5% CO_2_ humidified air in Dulbecco′s Modified Eagle′s Medium (DMEM) without phenol red supplemented with 10% dialyzed fetal bovine serum, 1% l-glutamine, 1% penicillin and streptomycin, and 50 µg/mL gentamycin.

ROS production in SH-SY5Y cell line was detected using Cellular Reactive Oxygen Species Detection Assay Kit (red fluorescence; Abcam, Cambridge, UK). Cells were seeded into clear polystyrene flat-bottomed 384-well microplates in culture medium without phenol red and containing dialyzed fetal bovine serum (volume 25 µL) at a plating density of 12,000 cells per well. After seeding, microtiter plates were incubated overnight at 37 °C prior to addition of the compounds. Phenelzine sulfate was used as positive control to evaluate the decrease of ROS production, while dopamine HCl (final conc. 10 nM) was used as a positive ROS inductor. The next day phenelzine sulfate and dantrolene sodium (final conc. 100 nM) were added and the plate was incubated at 37 °C and 5% CO_2_ for 45 min (final volume 50 µL). The dilutions of the compounds and their addition was performed by liquid handler ECHO 550 (Labcyte Ltd, Cannock, UK). After 45 min the cellular medium was removed and 25 µL of dopamine were added to all the wells, except for those assigned to the negative control, and incubated for 10 min. Subsequently the dopamine solution was removed, a washing with commercial PBS was performed and 25 µL of DCFDA solution (25 µM) were added. The plate was incubated at 37 °C and 5% CO_2_ for 35 min in the dark. The DCFDA solution was then eliminated and a further washing with commercial PBS was performed. After washing, the fluorescence was read immediately, and the readings were taken every 15 min in a 60-min time interval. Fluorescence signal at 535 nm (excitation at 485 nm) was measured using Tecan Infinite M1000 Pro (Tecan, Mannedorf, Switzerland).

### 3.8. CAC Activation Assay

#### 3.8.1. Preparation of Rat Liver Mitochondria and Carnitine/Acylcarnitine Carrier (CAC) Transport Activity Measurement in Entire Organelle

In order to isolate crude mitochondria from rat liver, a standard protocol was performed, involving cell destruction by mechanical stress, isolation by differential centrifugation in order to eliminate various subcellular contaminations, and purification by centrifugation on colloidal silica Percoll 30% gradient [54]. The average purity of the mitochondrial preparation was monitored by measuring the specific activity of alkaline phosphatase (EC 3.1.3.1). The specific activity of alkaline phosphatase, in the mitochondria preparations was measured at 37 °C as substrate consumed per min per mg protein [55]. The measurement of CAC activity in isolated entire mitochondria has been performed as previously described [56], except for some variations. The purified mitochondria (5 mg/100 μL) were incubated for 2 h with 20 mM carnitine, washed three times with a buffer containing 150 mM sucrose, 50 mM TRIS-HCl, 50 mM KCl, and the transport activity was measured. The uptake was started by adding 0.05 mM [^3^H]-carnitine (Scopus Research B.V., Wageningen, Netherlands) and the transport activity was inhibited at various times (inhibitor-stop method) [57] by a series of washings with the same buffer used before adding cold 1mM NEM. Finally, the samples were read by a liquid scintillation counter.

#### 3.8.2. Site-Directed Mutagenesis, Overexpression, and Isolation of the CAC Proteins

The previously constructed pMW7-WTrat CAC recombinant plasmid [58] was used as a template to introduce mutations in the CAC protein using complementary mutagenic primers [59] and the High Fidelity PCR System (Roche, Basel, Switzerland). The PCR products were purified by the QIAEX II Gel Extraction Kit (QIAGEN, Milan, Italy), digested with the restriction enzymes Nde I and Hind III (New England Biolabs, Ipswich, MA, USA) and ligated into the pMW7 cloning vector (given as a gift). All resulting pMW7-rat CAC constructs were verified by DNA sequencing. Except for the desired base changes, all the sequences corresponded to the CAC coding sequence. Bacterial overexpression was obtained using the expression vector of *Escherichia coli* C0214 (given as a gift). Wild-type and mutants rat CAC inclusion body fractions were isolated from *E. coli*, solubilized, and purified as previously described [58].

#### 3.8.3. Reconstitution of Mitochondrial Extract or Recombinant CAC Proteins in Liposomes

Mitochondrial rat liver extract (0.3 mg proteins in 3% Triton X-100) containing native CAC was reconstituted into liposomes by removing the detergent from the mixed micelles through a hydrophobic ion-exchange column containing the resin Amberlite XAD-4, as described previously [44]. The reconstitution mixture of mitochondrial or recombinant CAC proteins was composed of protein, 1% Triton X-100, 10 mg of egg yolk phospholipids (l-α-phosphatidylcholine from fresh turkey egg yolk), in the form of sonicated liposomes, 30 mM NaH_2_PO_4_ at pH 7.0, 15 mM carnitine, in a final volume of 680 μL. This mixture was passed 15 times, at room temperature, through the same Amberlite column (Pasteur pipette filled with 0.5 g resin) pre-equilibrated with a buffer containing 30 mM NaH_2_PO_4_ at pH 7.0 and 15 mM carnitine.

#### 3.8.4. Transport Measurements

Before starting the transport activity, proteoliposomes were passed through a Sephadex G-75 column (0.7 cm diameter; 15 cm height; Pharmacia, Milan, Italy) to remove the external substrate. The turbid eluate (550 μL) from the Sephadex column was collected and used for transport measurement by the inhibitor-stop method [57]. For uptake measurements, transport at 25 °C was started by adding 0.1 mM [^3^H]-carnitine to proteoliposomes and stopped by the addition of 0.1 mM NEM. In control samples, the inhibitor was added together with the labeled substrate at time zero. The experimental values were corrected by subtracting control values. Finally, the external substrate was removed by chromatography on Sephadex G-75 columns (0.7 cm diameter; 10 cm height) and intraliposomal radioactivity was measured by a liquid scintillation counter (Perkin Elmer, Milan, Italy).

#### 3.8.5. EC_50_ Calculation and Statistical Analysis

EC_50_ values were calculated using AATBioquest EC_50_ calculator [60]. Statistical analysis was performed by Student’s *t*-test, as indicated in figure legends. Values of *p* < 0.05 were considered statistically significant. Data points were derived from the mean of three different experiments, as specified in the figure legends.

## 4. Conclusions

Repurposing of existing drugs is considered a valid shortcut to alternative therapies or, as in the case of neurodegenerative diseases, to give chances to unmet therapeutic needs [61]. The main advantage of this approach is in avoiding the preclinical phase of drug development, thus minimizing the costs required for ADMET profiling and the business risks for companies and investors. On the other hand, the main challenge of drug repurposing is to achieve the desired off-label activities regardless of the original therapeutic effect or balancing it into a risk/benefit evaluation for a new therapeutic use. In this context, we disclosed new biological activities exerted by dantrolene, a drug specifically used in the management of malignant hyperthermia, which has been proposed for repurposing in several pathologies, including MDMA intoxication [8] and Duchenne muscular dystrophy [62]. In this work, we demonstrated for the first time that DAN competitively inhibits MAO B with a micromolar K_i_ value for the human enzyme, showing, in addition, activity as an AChE inhibitor and hampering the aggregation of Aβ_40_ and PHF6, i.e., two probes of amyloid aggregation in AD brain. Regarding the antioxidant properties [28], we showed that DAN, ineffective as a radical scavenger, decreases ROS production in a cell-based assay of neuroprotection from OS, this activity being apparently related to its MAO inhibitory activity.

A significant outcome of this work was the discovery of the activation of the carnitine/acylcarnitine carrier exerted by DAN, with an EC_50_ of 9.3 μM for the purified recombinant WT protein. This transporter acts through reductive activation and is involved in trafficking of acyl groups into the mitochondria, carried by l-carnitine. By treatment with DAN, this transport system ultimately facilitates, under OS conditions, the restoring of ATP production and thus cell vitality, and an increase of export from mitochondria of endogenous acetyl-l-carnitine, a well-known nutraceutical with neuroprotective effects [63]. Herein, we demonstrated that the activation of CAC exerted by DAN takes place via a redox mechanism involving the reduction of specific disulfide bridges of the protein. Such a feature, which appears in contrast with the lack of any intrinsic antioxidant activity, deserves further investigations.

Finally, DAN acts in the therapy of MH by inhibiting ryanodine receptors, which are responsible for Ca^2+^ recruitment from sarcoplasmic reticulum, and are hyperactivated in MH. Such on-target activity of DAN is in full accordance with the pleiotropic actions of DAN disclosed herein. Since calcium dyshomeostasis represents one of the main alterations in AD, the therapeutic potential of DAN has been suggested in cellular [64] and animal models of AD [65].

Taken together, all the biological activities provide a body of evidence that prompts the repurposing of DAN as a multitarget neuroprotective agent, with potential in AD to be challenged with in vivo animal models of neurodegeneration.

## Figures and Tables

**Table 1:** Enzyme inhibition data (μM) for dantrolene and reference drugs pargyline and galantamine for MAO and ChE inhibition, respectively. Values are mean ± SEM of three independent experiments.

	hMAO B		hMAO A	hAChE		hBChE
	IC_50_	K_i_	IC_50_	IC_50_	K_i_	IC_50_
Dantrolene	2.69 ± 0.44	0.96 ± 0.09 (comp.)	14.0 ± 1.0	4.19 ± 0.73	6.30 ± 0.15 (noncomp.)	No inhibition
Pargyline	2.69 ± 0.48	-	10.9 ± 0.6	-	-	-
Galantamine	-	-	-	0.62 ± 0.13	-	8.78 ± 0.36
